# Gynoecious and monoecious cucumbers drive the assembly of different rhizosphere microbial communities

**DOI:** 10.3389/fpls.2026.1786995

**Published:** 2026-03-06

**Authors:** Liyuan Liao, Xinyan Zhou, Xinni Li, Yan Yin, Ken Chen, Simeng Liu, Shangdong Yang

**Affiliations:** Guangxi Key Laboratory of Agro-environment and Agro-products Safety, National Demonstration Center for Experimental Plant Science Education, College of Agriculture, Guangxi University, Nanning, Guangxi, China

**Keywords:** cucumber (*Cucumis sativus L.*), plant-microbe interaction, rhizosphere, sex expression, soil microbial community structure

## Abstract

Cucumber sex expression is a key agronomic trait determining yield, but whether its formations is related to rhizosphere soil microbes remains poorly understood. This study compared the soil microbial community structures in rhizosphere between gynoecious and monoecious cucumbers to identify potential associations. The results showed that bacterial genera including *Sphingomonas*, and other unclassified taxa, were significantly enriched in the rhizosphere of the gynoecious plants. In contrast, members of Rokubacteriales and other taxa were significantly enriched in rhizosphere of monoecious cucumbers. For fungi, genera such as *Aspergillus*, *Plectosphaerella*, and *Chaetomella* were enriched in rhizosphere of gynoecious plants. Conversely, *Trichoderma*, *Emericellopsis*, *Collariella*, and *Cordana* were significantly enriched in monoecious cucumbers. Correlation network analysis revealed that the rhizosphere microbial network (especially the bacterial community) was more stable and displayed greater interspecific cooperation in monoecious cucumbers. Functional prediction revealed that multiple nitrogen-cycling processes of bacterial communities, including nitrification, aerobic nitrite oxidation, nitrite and nitrate ammonification, aerobic ammonia oxidation, and arsenate respiration were detected in rhizosphere of the gynoecious cucumbers. By contrast, hydrocarbon degradation functions, particularly those for aromatic and aliphatic non-methane hydrocarbons were significantly enriched in rhizosphere of monoecious cucumbers. Moreover, the rhizosphere of gynoecious plants harbored a higher abundance of saprotrophic and symbiotrophic fungi but a lower abundance of pathotrophic fungi compared with monoecious cucumbers. These findings demonstrate that the composition and potential functions of the rhizosphere microbiota differ between gynoecious and monoecious plants, indicating that soil microbes in rhizosphere play a role in the sex expression of cucumber varieties.

## Introduction

Cucumber (*Cucumis sativus* L.) exhibits diverse sex expressions, with gynoecious and monoecious being two representative phenotypes (Dhall et al., 2023). Being a critical agronomic trait, sex expression directly influences fruit yield and cultivation efficiency. For instance, gynoecious plants incur higher resource costs for fruit production compared with male plants (Lei et al., 2017). Moreover, different sex types vary in their responses to environmental stresses (Liu et al., 2021). Notably, gynoecious cultivars often demonstrate lower abiotic stress tolerance than their monoecious counterparts (Miao et al., 2011).

Cucumber sex determination is governed by an interactive network of genetic, hormonal, and environmental factors. The genetic framework is primarily controlled by three loci: *F* (promoting female flower), *M* (essential for stamens), and *A* (influencing hermaphroditic flowers) (Pan et al., 2018). Endogenous hormones interact with genetic instructions, with ethylene and gibberellins playing central, antagonistic roles. Ethylene acts as a key feminizing hormone: its biosynthetic gene *CsACS2*, regulated by the *F* locus, initiates local pistil development (Zhang et al., 2017; Saito et al., 2007). Conversely, gibberellins predominantly promote male differentiation (Adhikari et al., 2012). Environmental factors such as photoperiod (Yamasaki et al., 2003), temperature (low night temperatures promote femaleness), and climate change (Nguyen et al., 2024; Aparna et al., 2023; Miao et al., 2011) further fine-tune sex expression by modulating this hormonal balance.

Plant-microbe interactions are now recognized as central in plant physiology. The plant microbiota, a crucial symbiotic system, significantly influences plant growth, defense, and health across different environments (Liu et al., 2025; Zhang et al., 2024; Sahu et al., 2019; Ortíz-Castro et al., 2009). Consequently, the effect of the plant, particularly of the rhizosphere, over microbial assembly may represent a key adaptive trait. The rhizosphere microbiome, known as the plants “second genome” (Berendsen et al., 2012), profoundly affects host growth and development by sustaining nutrient acquisition, hormone regulation, and pathogen resistance (Jiang et al., 2024; Lan et al., 2024; Ping et al., 2024; Klikno and Kutschera, 2017). Host genotype and developmental stage affect the composition of root exudates, the primary mediators of plant-microbe interactions (Anderson et al., 2024), which in turn structures the rhizosphere microbial community (Hu et al., 2018). Moreover, microbes can influence floral development by disrupting hormone homeostasis (e.g., via indole-3-acetic acid production) or mimicking plant signals (Lu et al., 2018). However, in cucumber, whether and how sex type shapes the rhizosphere microbiome, and whether these microbes provide feedback to influence sex differentiation, remains unknown. By comparing the rhizosphere microbial communities of gynoecious and monoecious cucumbers, this study aims to uncover these differences, advancing our understanding of plant-microbe interactions.

## Materials and methods

### Experimental site overview and plant materials

The experiment was conducted from March to May 2024 at the Vegetable Teaching and Research Base, College of Agriculture, Guangxi University (108°17′25″E, 22°51′02″N). The soil at the experimental site is classified as lateritic red earth, with the following physicochemical properties: pH 5.64, organic matter 23.26 g·kg^-^¹, total contents of nitrogen, phosphorus and potassium were 1.22, 0.57 and 6.8 g·kg^-^¹, respectively. Meanwhile, the contents of available nitrogen, phosphorus and potassium were 15.6, 0.73 and 83.3 mg·kg^−1^, respectively.

Three gynoecious cucumber cultivars, ‘Ouluoba Yihao’, ‘Meicui 808’, and ‘Shuiguo Huanggua F1’, and three monoecious cultivars, ‘Zhencui 101’, ‘Tiancui’, and ‘Zhenhaochi Jiejiegua’, were used in this study. The three gynoecious cultivars produced only female flowers at each node, with high and stable fruit set rate, smooth and uniform fruits, and good adaptability to protected cultivation. In contrast, the three monoecious cultivars produced male and female flowers sequentially, exhibited vigorous vegetative growth and strong heat tolerance, and set fruits predominantly on the main vine. All experimental materials were F_1_ hybrids, and seed quality conformed to Chinese national standards (purity ≥ 95.0%, cleanliness ≥ 99.0%, germination rate ≥ 85%, moisture content ≤ 8.0%). Seeds were sown in the experimental year, and all plants were cultivated under uniform management conditions.

### Experimental design and cultivation management

Cucumber seeds were germinated in late March 2024. Seedlings were transplanted to the field in April following a randomized complete block design (RCBD) with three replicates per cultivar. Rhizosphere soil samples were collected in May. All plots received uniform management, including standard irrigation, weeding, and pest control practices.

### Rhizosphere soil sampling

Rhizosphere soil samples were collected from three randomly selected plants per treatment. Using a sterilized spatula, the soil around each plant was gently loosened before the plant was carefully uprooted. The soil tightly adhering to the roots, defined as the rhizosphere fraction (Zhou et al., 2024), was collected. Additionally, soil samples from the same experimental field without growing cucumbers were also randomly collected as background (CK) samples. All samples were immediately placed in pre-labeled, sterile zip-lock bags and stored on ice during transport to the laboratory. There, coarse debris was removed, and the soil was sieved through a 2 mm mesh to achieve homogenity for subsequent microbial community analysis (Ling et al., 2015). This sampling protocol resulted in three biological replicates per cultivar.

### DNA extraction, PCR amplification, and high-throughput sequencing

Total genomic DNA was extracted from cucumber rhizospheric soil samples using the FastDNA^®^ Spin Kit for Soil (MP Biomedicals, USA) following the manufacturer’s protocol. DNA concentration and purity were verified using NanoDrop2000 spectrophotometer (Thermo Fisher Scientific, USA). We then amplified the target regions (for primer details, see **Table 1**) via Polymerase chain reaction (PCR) amplification on an ABI GeneAmp^®^ 9700 thermal cycler. The resulting amplicons were purified with the AxyPrep DNA Gel Extraction Kit (Axygen Biosciences, USA), combined in equimolar ratios, and quality-checked through 2% agarose gel electrophoresis. The pooled library was quantified with a Quantus™ Fluorometer (Promega, USA) and prepared for sequencing using the NEXTFLEX^®^ Rapid DNA-Seq Kit.

**Table 1:** Primer names and sequences.

Primer type	Primer name	Primer sequence (5’-3’)	Sequencing platform	Sequencing length(bp)
Rhizosphere bacteria	338F	ACTCCTACGGGAGGCAGCAG	PE300	416
	806R	GGACTACHVGGGTWTCTAAT		
Rhizosphere fungi	ITS1F	CTTGGTCATTTAGAGGAAGTAA	PE300	242
	ITS2R	GCTGCGTTCTTCATCGATGC		

All laboratory procedures, including DNA extraction, PCR, and library preparation, were conducted by Shanghai Majorbio Bio-Pharm Technology Co., Ltd. Finally, high-throughput sequencing was performed on the Illumina MiSeq PE300 platform (Illumina, USA) to generate 2×300bp paired-end reads.

### Bioinformatic processing and statistical analysis

Raw FASTQ files from 18 samples were demultiplexed according to unique barcodes using a custom Perl script. Quality control was performed with fastp (v0.19.6), and paired-end reads were assembled using FLASH (v1.2.7). Reads were truncated when the mean quality score within a 50-bp sliding window fell below 20. Sequences shorter than 50 bp after trimming, containing ambiguous bases, or that could not be assembled with an overlap >10 bp and mismatch rate <20% were discarded. Exact barcode matching and up to 2 primer mismatches were allowed for sample assignment, and read orientation was corrected before downstream processing.

High-quality sequences were clustered into operational taxonomic units (OTUs) at a 97% sequence similarity using UPARSE (v7.1), and the most abundant sequence within each OTU was selected as the representative. An OTU-based framework (rather than amplicon sequence variant, ASV, inference) was adopted to maintain methodological consistency with prior cucumber rhizosphere microbiome studies and to enable cross-database ecological comparability. As our objective was to characterize overall community structure rather than transcriptionally active populations, total bulk community DNA was analyzed rather than RNA. While relic DNA from dormant or dead cells may be present, DNA-based amplicon profiling remains the most widely adopted and comparable approach in soil microbial community ecology.

To minimize sequencing noise, singleton OTUs and extremely low-abundance taxa were excluded prior to diversity and community composition analyses. Taxonomic annotation of representative sequences was performed using the RDP Classifier (v2.11): sequences were aligned against the SILVA 16S rRNA database (release 138) for bacteria and the UNITE database (release 8.0) for fungi, with a confidence threshold of 0.7. Bacterial nomenclature was further validated for consistency against the List of Prokaryotic names with Standing in Nomenclature (LPSN) where appropriate.

Alpha-diversity indices (Shannon, Simpson, ACE, and Chao1) were calculated in Mothur (v1.30.2). Inter-group comparisons of alpha-diversity indices were performed using the Wilcoxon rank-sum test in R (v4.3.3), with a significance threshold of *P* < 0.05. Beta diversity was assessed using Bray–Curtis dissimilarity computed with the vegdist function in the vegan package (v2.6-4.6) in R (v4.3.3). Principal coordinates analysis (PCoA) was carried out using the ape package (v5.7-1.7) and visualization with ggplot2 (v3.4.4). Group separation was evaluated by partial least squares discriminant analysis (PLS-DA) implemented in mixOmics (v6.26.0). Taxa showing differential abundance from phylum to genus level were identified using LEfSe, with thresholds of LDA > 3.0 and *P* < 0.05. Putative functional profiles of bacterial and fungal communities were inferred using FAPROTAX (v1.2.1) and FUNGuild, respectively.

## Results

### Soil bacterial and fungal alpha diversity in rhizosphere

All samples exhibited high Coverage values (exceeding 96%, **Table 2**), confirming that the sequencing depth was sufficient to capture the majority of microbial species, and that the data reliably reflect the true diversity and richness of the communities. For bacterial communities, the Shannon index showed no significant difference, as also the Simpson index. Meanwhile, the richness indices (Ace and Chao1) also revealed no significant difference between the rhizosphere soils of gynoecious and monoecious cucumbers, but both differed significantly from the bulk soil. Additionally, for fungal communities, neither the diversity (Shannon and Simpson) nor richness (Ace and Chao1) indices showed significant differences among treatments.

**Table 2:** Alpha-diversity indices of the rhizosphere microbiota between gynoecious and monoecious cucumbers.

Category	Treatment	Shannon index	Simpson index	Ace index	Chao1 index	Coverage
Rhizosphere bacteria	Gynoecious	7.08 ± 0.05a	0.003 ± 0.0006a	5040 ± 177.7b	4770 ± 168.3b	0.96
	Monoecious	7.06 ± 0.08a	0.003 ± 0.0004a	5083 ± 268.4b	4818 ± 235.8b	0.96
	CK	7.18 ± 0.07a	0.003 ± 0.0002a	5621 ± 151.6a	5281 ± 108.8a	0.96
Rhizosphere fungi	Gynoecious	4.45 ± 0.14a	0.03 ± 0.01a	919 ± 49.14a	925 ± 50.15a	0.99
	Monoecious	4.17 ± 0.42a	0.06 ± 0.04a	855.7 ± 134.2a	857.5 ± 133.1a	0.99
	CK	4.36 ± 0.11a	0.04 ± 0.006a	858.9 ± 54.96a	864.3 ± 58.96a	0.99

Data shown are mean ± standard deviation. Different letters in the same column indicate significant differences among the treatments at *P* < 0.05.

### Soil bacterial and fungal beta diversity in rhizosphere of gynoecious and monoecious cucumbers

**Figure 1:**
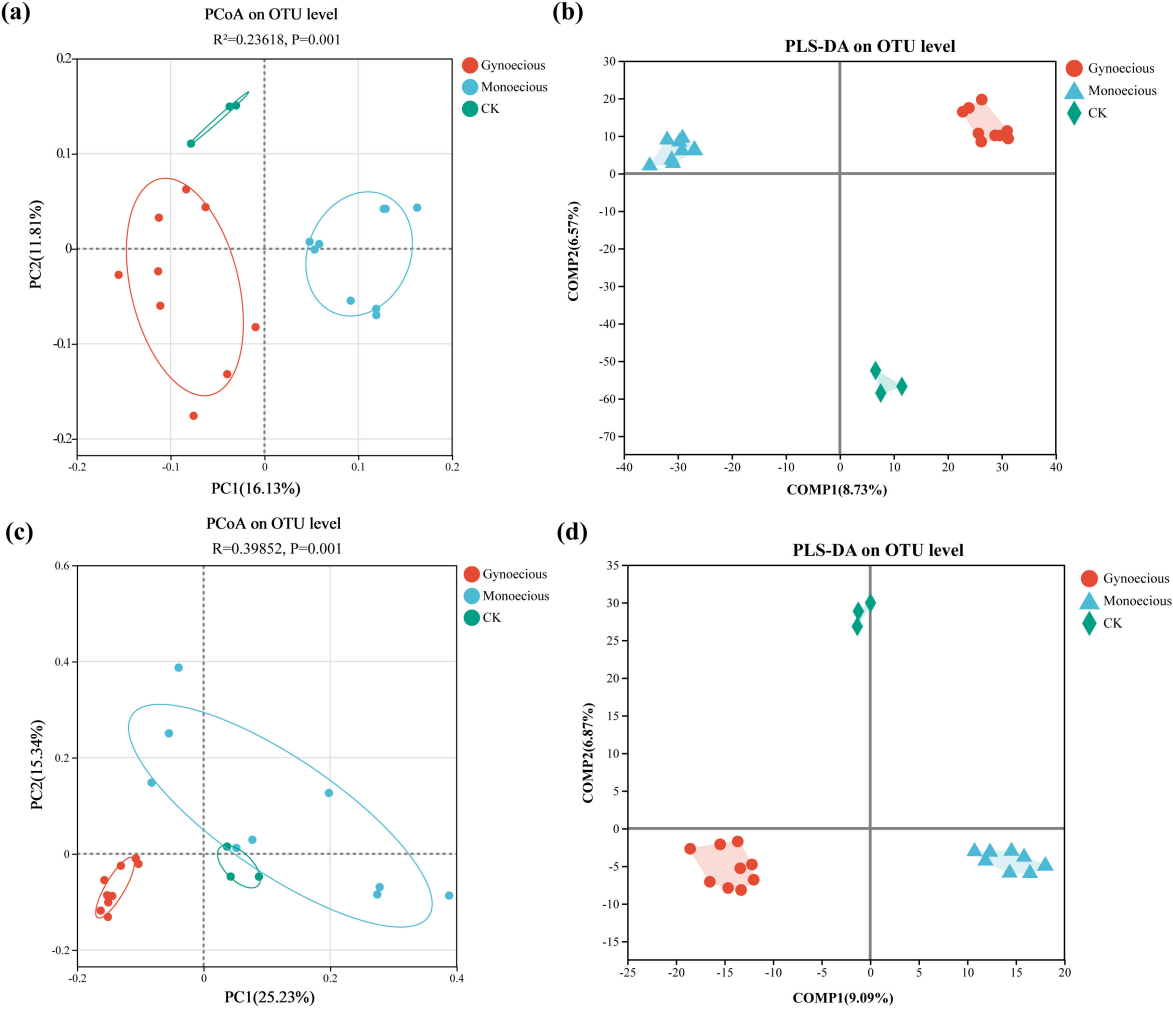
Comparative analysis of microbial communities in the rhizosphere of gynoecious and monoecious cucumbers versus bulk soil (CK). **(A)** PCoA of rhizosphere bacterial communities at the OTU level. **(B)** PLS-DA score plot of rhizosphere bacterial communities. **(C)** PCoA of rhizosphere fungal communities at the OTU level. **(D)** PLS-DA score plot of rhizosphere fungal communities.

We used Principal Coordinates Analysis (PCoA) based on Bray-Curtis distances to visualize differences in OTU-level soil bacterial and fungal community structures, which were statistically tested using PERMANOVA (Adonis). The analysis revealed significant structural differences for both communities. For bacterial communities, the Adonis test produced significant results (R² = 0.2362, *P* = 0.001; **Figure 1A**), indicating that the variation between treatments was substantially greater than within them. A more pronounced effect was observed for fungal communities (R² = 0.3985, *P* = 0.001; **Figure 1C**). Furthermore, the OTU-level compositions of both bacterial and fungal compositions differed significantly between the gynoecious and monoecious cucumber lines.

These patterns were supported by Partial Least Squares Discriminant Analysis (PLS-DA) that showed a clear separation among OTU-based bacterial communities from the gynoecious cucumbers, monoecious cucumbers, and the bulk soil (CK; **Figure 1B**), indicating that cucumber lines with different sex types assemble distinct bacterial consortia in their rhizospheres. A similar clustering pattern was observed for the fungal communities (**Figure 1D**), suggesting that the host sex type also influences the assembly of root-associated fungal communities at the OTU level.

### Soil bacterial and fungal communities in the rhizosphere of gynoecious and monoecious cucumbers

**Figure 2:**
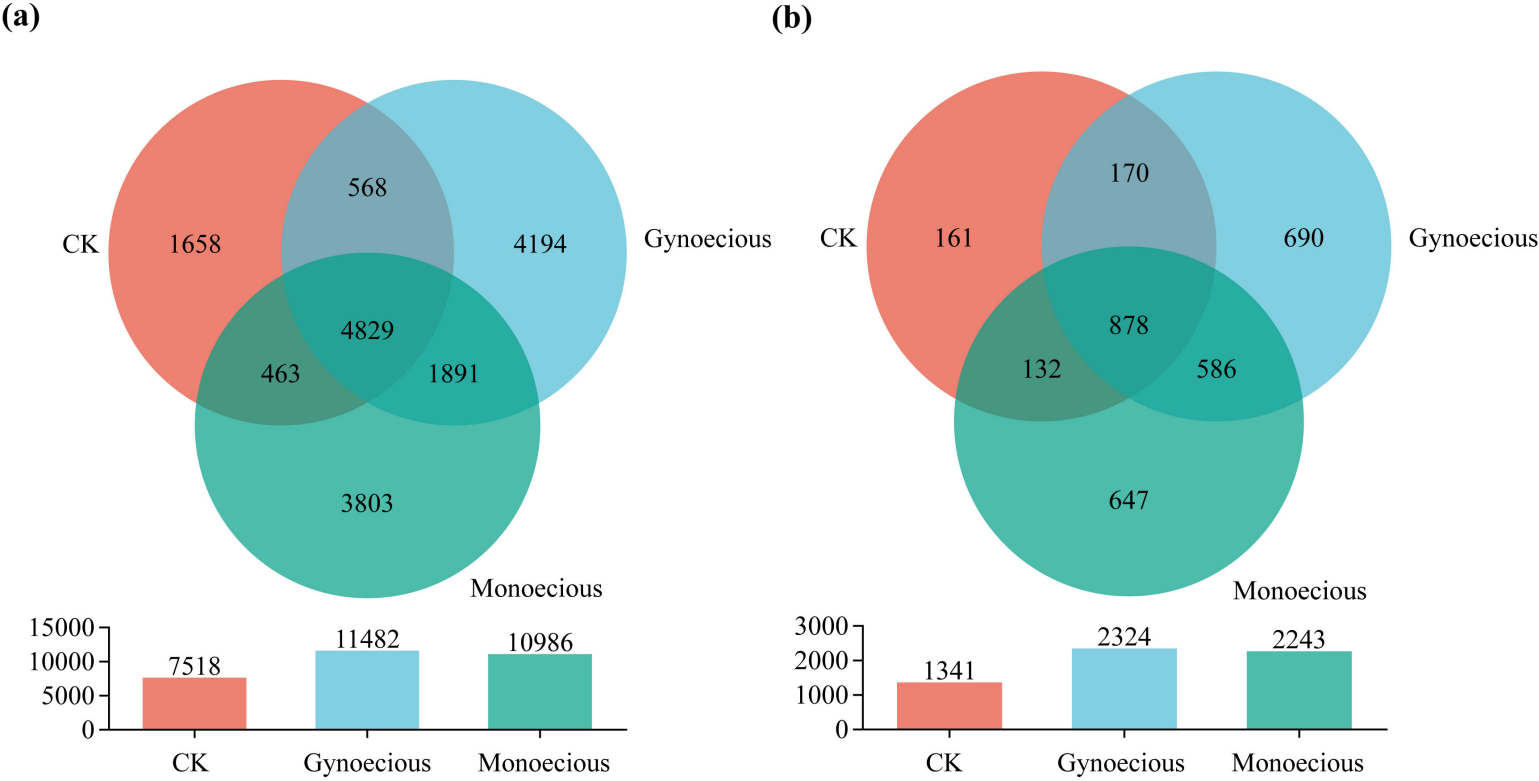
Venn diagram of bacterial **(A)** and fungal **(B)** communities in the rhizosphere of gynoecious and monoecious cucumbers.

Venn analysis at the OTU level revealed a substantial number of unique bacterial and fungal OTUs in the rhizosphere of each cucumber line (**Figure 2**). Specifically, the gynoecious and bulk soil (CK) showed the highest number of OTUs, followed by momoecious (**Figure 2A**). A similar trend was observed for fungi (**Figure 2B**).

### Distinct microbial community structures in rhizosphere of gynoecious and monoecious cucumbers

**Figure 3:**
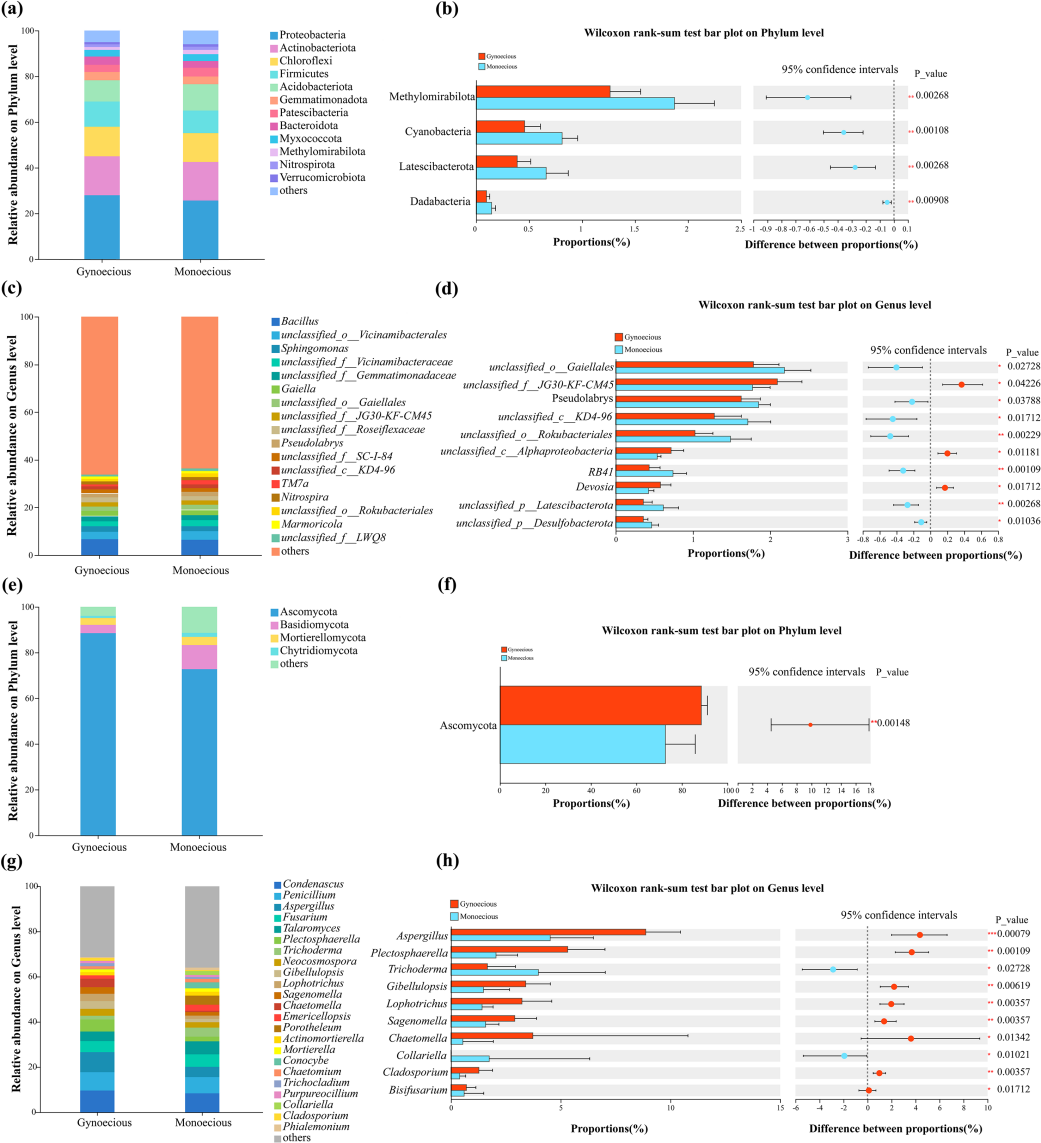
Microbial composition in the rhizosphere of gynoecious and monoecious cucumbers at phylum and genus levels. **(A)** Bar plot showing relative abundance of bacterial communities at the phylum level. **(B)** Significantly different bacterial phyla between gynoecious and monoecious cucumbers (Wilcoxon rank-sum test). **(C)** Bar plot showing relative abundance of bacterial communities at the genus level. **(D)** Significantly different bacterial genera between the two cultivars (Wilcoxon rank-sum test). **(E)** Bar plot showing relative abundance of fungal communities at the phylum level. **(F)** Significantly different fungal phyla between the two cultivars (Wilcoxon rank-sum test). **(G)** Bar plot showing relative abundance of fungal communities at the genus level. **(H)** Significantly different fungal genera between the two cultivars (Wilcoxon rank-sum test). *0.01 < *P* ≤ 0.05; **0.001 < *P* ≤ 0.01; ***P ≤ 0.001.

Comparative analysis revealed differences in the dominant microbial taxa between gynoecious and monoecious cucumbers rhizospheres. The rhizosphere of gynoecious and monoecious cucumbers harbored 11 and 12 dominant bacterial phyla (relative abundance > 1%), respectively. While the top three phyla (Proteobacteria, Actinobacteriota, and Chloroflexi) were consistent between lines, Verrucomicrobiota was uniquely dominant in the monoecious line (**Figure 3A**).

At the genus level, 16 and 17 dominant bacterial genera were identified in the gynoecious and monoecious lines, respectively. *Unclassified_f:LWQ8* was a unique dominant genus in the monoecious lines, whereas the gynoecious lines lacked any unique dominant bacterial genus (**Figure 3C**). The top five most abundant genera in the gynoecious line were *Bacillus*, *unclassified_o:Vicinamibacterales*, *Sphingomonas*, *unclassified_f_*_*Gemmatimonadaceae*, and *Gaiella.* Wilcoxon rank-sum tests confirmed significant differences in the abundance of four bacterial phyla (**Figure 3B**) and ten genera (**Figure 3D**).

The fungal compositions also differed. We detected four and five dominant fungal phyla in the gynoecious and monoecious rhizospheres, respectively (**Figure 3E**). At the genus level, although 19 dominant fungal genera were identified in both lines, their composition also differed. The genera *Chaetomella*, *Trichocladium*, *Purpureocillium*, and *Cladosporium* were unique to the gynoecious rhizosphere, while *Porotheleum*, *Conocybe*, *Collariella*, and *Phialemonium* were unique to the monoecious rhizosphere (**Figure 3G**). Wilcoxon rank-sum tests revealed significant differences in one fungal phylum (**Figure 3F**) and ten fungal genera (**Figure 3H**).

We performed LEfSe analysis (LDA threshold>3.0) to identify biomarker taxa that were significantly enriched in the rhizosphere of either gynoecious and monoecious cucumbers. The resulting LDA scores reflect the effect size of each taxon’s contribution to the differences between treatments.

**Figure 4:**
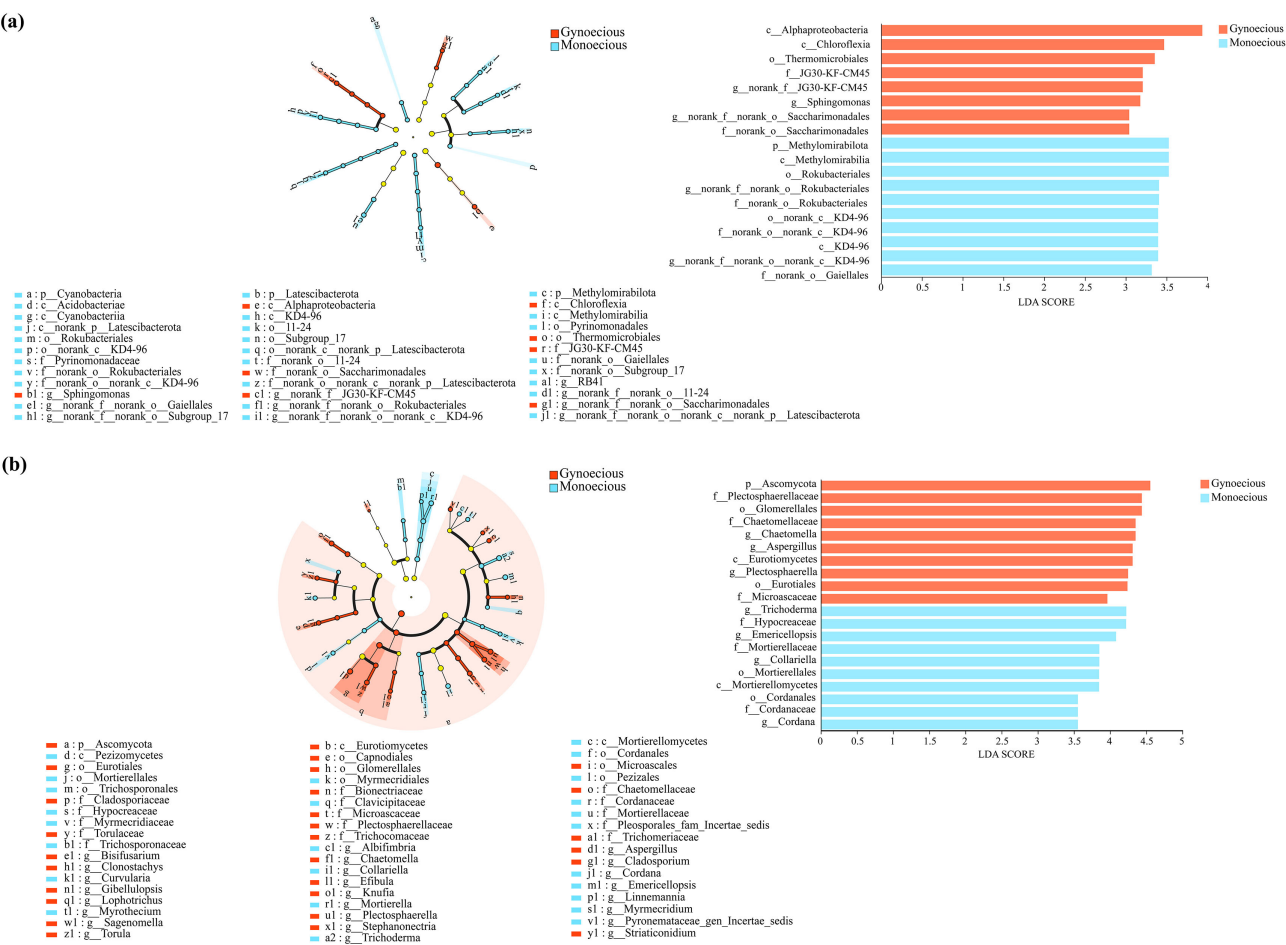
LEfSe analysis identifying differentially enriched taxa in the rhizosphere bacterial **(A)** and fungal **(B)** communities between gynoecious and monoecious cucumbers (LDA > 3.0).

At the bacterial phylum level, Methylomirabilota was a significant biomarker for the monoecious cucumber rhizosphere. At the bacterial genus level, *Sphingomonas*, *unclassified*_*f_*_*JG30*-*KF*-*CM45*, and *unclassified*_*o_*_*Saccharimonadales* were significantly enriched in the gynoecious cucumber rhizosphere. In contrast, *unclassified_c_*_*KD4–96* and *unclassified_o:Rokubacteriales* were significantly enriched in the monoecious cucumber rhizosphere (**Figure 4A**).

For the fungal communities, the phylum Ascomycota was significantly enriched in the gynoecious cucumber rhizosphere. Several genera served as key biomarkers: *Aspergillus*, *Plectosphaerella*, and *Chaetomella* were associated with the gynoecious rhizosphere, while *Trichoderma*, *Emericellopsis*, *Collariella*, and *Cordana* were significantly distinctive biomarkers for the monoecious cucumber rhizosphere (**Figure 4B**).

**Figure 5:**
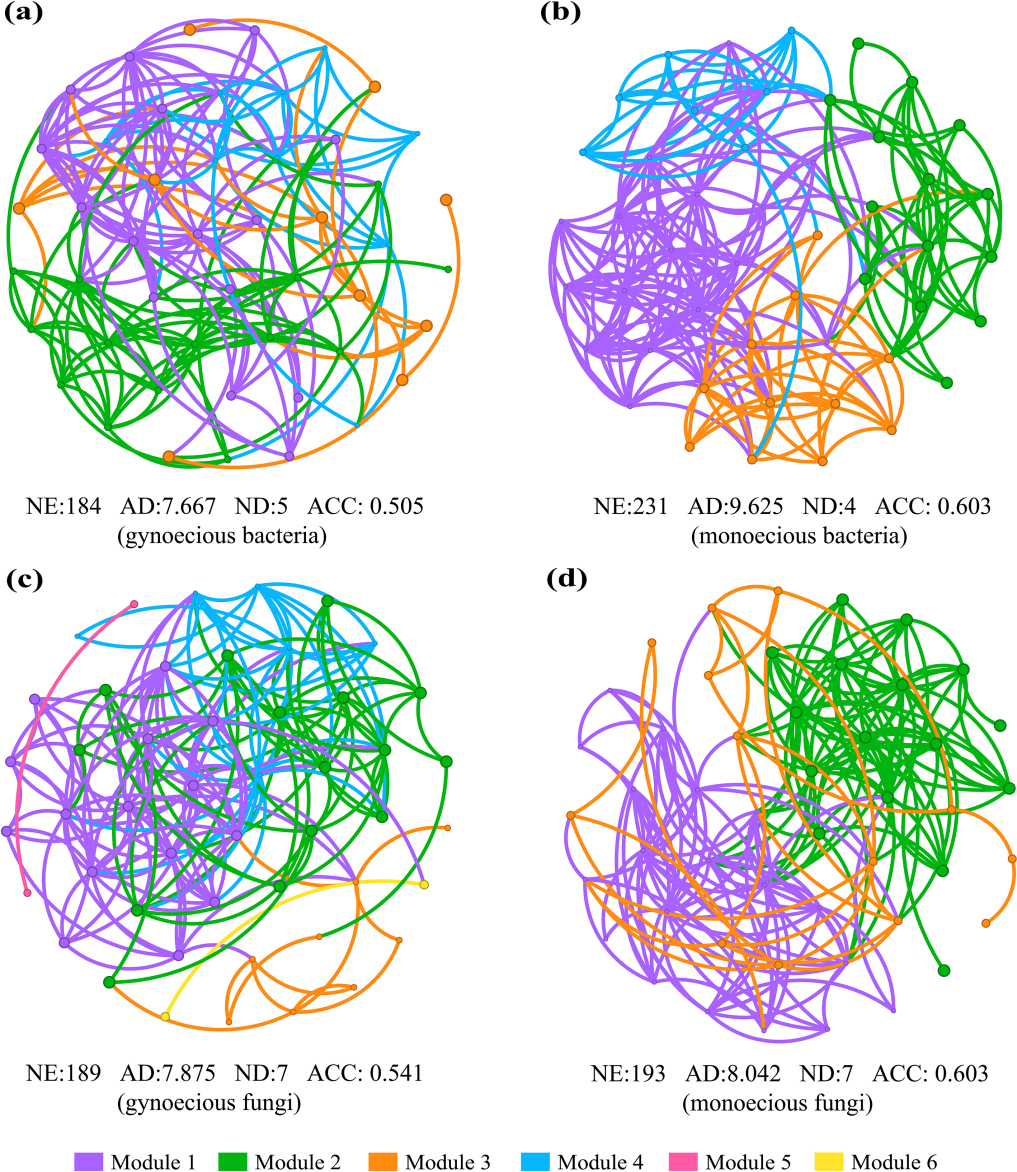
Co-occurrence networks of rhizosphere bacterial communities in gynoecious **(A)** and monoecious **(B)** cucumber plants, and fungal communities in gynoecious **(C)** and monoecious **(D)** cucumber plants. Each node corresponds to one genus, with different colors representing different modules. NE, numbers of edges; AD, average degree; ND, network diameter; ACC, average clustering coefficient.

To investigate interspecific relationships among rhizosphere soil microorganisms in cucumber genotypes with contrasting sexual phenotypes, pairwise correlation network analysis was performed based on Spearman’s correlation coefficient (ρ ≥ 0.5, *P* < 0.05). For bacterial network, monoecious plants had a greater number of edges (NE = 231), higher average degree (AD = 9.625), and average clustering coefficient (ACC = 0.603) than gynoecious plants (NE = 184, AD = 7.667, ACC = 0.505), while exhibiting a smaller network diameter (ND = 4) compared with gynoecious plants (ND = 5) (**Figures 5A, B**). A similar pattern was observed in fungal networks: monoecious plants displayed more edge numbers (NE = 193), a higher average degree (AD = 8.042), and a higher average clustering coefficient (ACC = 0.603) relative to gynoecious plants (NE = 189, AD = 7.875, ACC = 0.541), with both groups sharing an identical network diameter (ND = 7) (**Figures 5C, D**). These results suggest that rhizosphere soil microbial networks—particularly bacterial communities—of monoecious plants possess greater structural stability and interspecific cooperativity than those of gynoecious plants.

### Functional prediction of the rhizosphere soil microbial community

**Figure 6:**
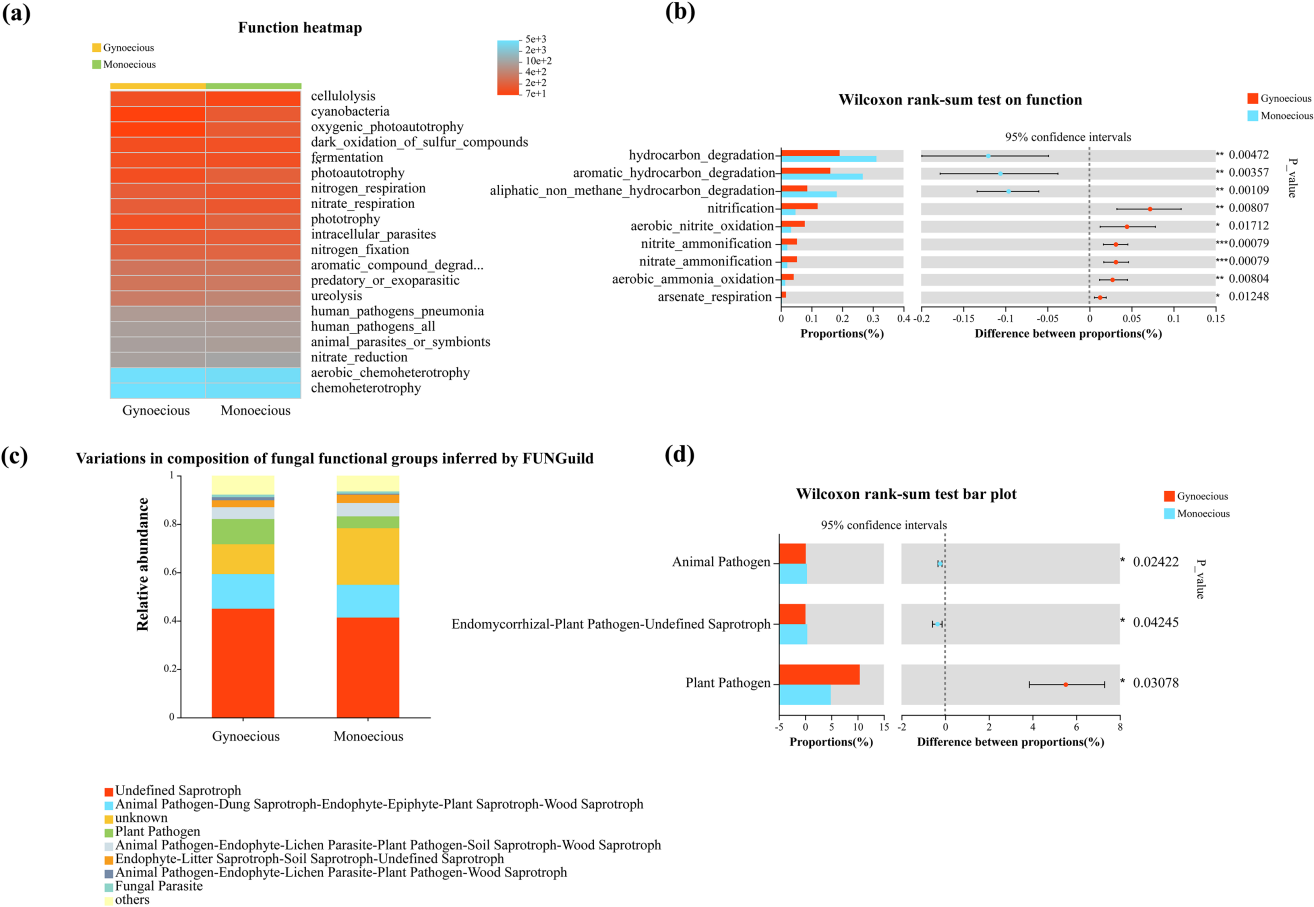
Functional prediction of rhizosphere microbial communities in gynoecious and monoecious cucumbers. **(A)** Bacterial functional potential inferred using FAPROTAX. **(B)** Bacterial functions with significantly different abundances. **(C)** Fungal trophic mode assigned by FUNGuild. **(D)** Fungal guilds with significantly different relative abundances. Differences were assessed with the Wilcoxon rank-sum test.

Using the FAPROTAX database, we predicted distinct metabolic potentials in the bacterial communities of gynoecious and monoecious lines (**Figure 6A**). Wilcoxon rank-sum tests identified key functions with significantly different abundances (**Figure 6B**). The gynoecious line was significantly enriched in multiple nitrogen-cycling processes, such as nitrification, aerobic nitrite oxidation, nitrite and nitrate ammonification, aerobic ammonia oxidation, and arsenate respiration. In contrast, the monoecious line showed significant enrichment in various hydrocarbon degradation functions, including those for aromatic and aliphatic non-methane hydrocarbons (**Figure 6B**).

For fungal communities, functional guilds were assigned using FUNGuild (**Figure 6C**), revealing significant differences in trophic modes. The monoecious lines exhibited a significantly higher relative abundance of pathotrophic fungi, while the gynoecious lines were enriched in both saprotrophic and symbiotrophic fungi (**Figure 6D**).

## Discussions

Plant sex differentiation is a complex process regulated by genetic, hormonal, and environmental factors. In recent years, increasing evidence has demonstrated that plant-associated microorganisms are also key regulators of plant growth and development (Garcia et al., 2024; Liu et al., 2021). Our study reveals distinct rhizosphere microbial communities and functional profiles between monoecious and gynoecious cucumbers, indicating that the microbiota may influence sex differentiation through hormone metabolism and nutrient supply pathways. Our findings suggest a potential intrinsic link between microbial community assembly and plant development.

Although α-diversity of both bacterial and fungal communities did not differ significantly between the two sexual types, β-diversity analyses (PCoA) and PLS-DA revealed clear separations in community structure. This indicates that cucumber sexual phenotype may exert selective pressure on rhizosphere microorganisms, likely through differences in root exudation and nutrient uptake patterns, thereby shaping sex-specific microbial assemblages. Similar sex-dependent microbial differentiation has been reported in *Populus* spp., *Carica papaya* L., and other dioecious or hermaphroditic plants (Zhu Q. L., et al., 2022; Zhou et al., 2022). These sex-specific microbiota not only reflect host-driven ecological niche differentiation but also imply a potential for feedback regulation, where microbial metabolic activities, in turn, could influence host reproductive development and performance.

The rhizosphere of monoecious cucumbers was significantly enriched in bacteria belong to Methylomirabilota, KD4–96 lineage (Chloroflexi), and “Candidatus Rokubacteria”. These taxa are known for their metabolic capabilities in methane oxidation, nitrate reduction, and organic matter decomposition (Freches and Fradinho, 2024; Zhu B., et al., 2022; Ivanova et al., 2022; Ettwig et al., 2010), which may regulate rhizosphere redox conditions and enhance nitrogen availability. Elevated nitrogen metabolism activity can promote gibberellin (GA) biosynthesis (Camut et al., 2021), a hormone that stimulates male flower development while inhibiting female flower differentiation (Zhang et al., 2014). Therefore, the enrichment of these bacteria might sustain higher GA levels in monoecious plants by reinforcing nitrogen cycling and GA synthesis pathways, thus favoring male organ development. Additionally, the monoecious rhizosphere was enriched with antagonistic fungi such as *Trichoderma* and *Emericellopsis*, which can secrete ethylene and cytokinins and induce host defense responses (Alburae et al., 2020; Contreras-Cornejo et al., 2016). These fungi may therefore play a pivotal role in balancing reproductive development and environmental adaptation.

Conversely, the rhizosphere of gynoecious cucumbers was dominated by *Sphingomonas*, Saccharimonadales, and fungal taxa belonging to Ascomycota (mainly *Aspergillus*, *Plectosphaerella*, and *Chaetomella*). Previous studies have shown that Ascomycota are relatively enriched in the rhizospheres of female plants (Guo et al., 2022). Both *Sphingomonas* and *Aspergillus* possess indole-3-acetic acid (IAA) biosynthesis capabilities and 1-aminocyclopropane-1-carboxylate (ACC) deaminase activity (Chauhan et al., 2024; Rahman et al., 2023; Mazoyon et al., 2023), which modulate the IAA–ethylene signaling pathway to promote female flower formation. Members of Saccharimonadales are closely associated with carbon metabolism and signal molecule synthesis (Figueroa-Gonzalez et al., 2020), potentially enhancing the diversity of root exudates and providing energy and signaling precursors for female flower development. These functions may synergistically create a rhizosphere microenvironment that favors ethylene and auxin signaling, facilitating femaleness.

Functional predictions further support a metabolic divergence between monoecious and gynoecious rhizospheres. According to FAPROTAX-based bacterial functional analysis, the gynoecious rhizosphere was significantly enriched in nitrogen oxidation-related pathways, including nitrification, aerobic nitrite oxidation, and aerobic ammonia oxidation. This suggests the presence of an active nitrogen-oxidizing metabolic network supplying energy and precursors for ethylene biosynthesis, since nitrogen availability can alter hormonal responses involving cytokinins and IAA (Xing et al., 2023). Conversely, the monoecious rhizosphere was enriched in hydrocarbon degradation and aromatic compound metabolism functions, which are related to carbon utilization and stress response. This pattern implies that its microbiome reflects energy balance and defensive metabolism to support the simultaneous development of both male and female floral organs.

Differences in fungal functional guilds also align with these roles in sex differentiation. FUNGuild analysis indicated that the gynoecious rhizosphere harbored higher proportions of saprotrophic and symbiotrophic fungi, which may facilitate organic matter decomposition and nutrient recycling, thereby improving carbon–nitrogen utilization efficiency and energy supply required for female flower development. In contrast, the monoecious rhizosphere exhibited a higher proportion of pathotrophic fungi, to face a defense burden, potentially altering hormonal equilibrium.

In summary, our findings suggest that the rhizosphere microbiota of gynoecious cucumbers may enhance ethylene and auxin signaling through nitrogen oxidation and hormone-promoting metabolic pathways, thereby facilitating female flower formation. In contrast, the microbiota associated with monoecious cucumbers tends to engage in hydrocarbon metabolism and stress-related functions, potentially maintaining GA-associated metabolism and defense balance to promote male flower development or sustain sexual coexistence. Collectively, the rhizosphere microbial community not only reflects the ecological adaptations of different sexual types but may also actively participate in the regulatory network of plant sex differentiation through metabolic and signaling interactions.

## Conclusions

Bacterial genera such as *Sphingomonas*, *unclassified*_*f_*_*JG30*-*KF*-*CM45*, and members of Saccharimonadales significantly enriched in the rhizosphere of gynoecious cucumber plants. In contrast, *unclassified_c_*_*KD4–96* and members of “Candidatus Rokubacteria” significantly enriched in rhizosphere of the monoecious cucumber. Additionally, fugal genera including *Aspergillus*, *Plectosphaerella*, and *Chaetomella* enriched in rhizosphere of gynoecious plants. Conversely, *Trichoderma*, *Emericellopsis*, *Collariella*, and *Cordana* significantly enriched in rhizosphere of monoecious cucumbers. The rhizosphere microbial co-occurrence network of monoecious cucumber plants, especially the bacterial community, displays greater complexity and stability relative to gynoecious plants. Functional prediction further revealed that multiple nitrogen-cycling processes of bacterial communities, such as nitrification, aerobic nitrite oxidation, nitrite ammonification, nitrate ammonification, aerobic ammonia oxidation, and arsenate respiration could be detected in rhizosphere of the gynoecious plants. In contrast, hydrocarbon degradation functions, including those for aromatic and aliphatic non-methane hydrocarbons were significantly enriched in rhizosphere of the monoecious cucumbers. Furthermore, the gynoecious cucumber rhizosphere harbored a higher relative abundance of saprotrophic and symbiotrophic fungi but a lower abundance of pathotrophic species compared to its monoecious counterpart. Collectively, these findings demonstrate that the composition and potential functions of the rhizosphere microbiota differ between gynoecious and monoecious plants, indicating that soil microbes in rhizosphere may play a role in the sex expression of cucumber varieties.

## Data Availability

The datasets presented in this study can be found in online repositories. The names of the repository/repositories and accession number(s) can be found in the article/supplementary material.
