# Supplementary figures and images for: Gynoecious and monoecious cucumbers drive the assembly of different rhizosphere microbial communities
Source: Front Plant Sci. 2026 Mar 6;17:1786995. doi: 10.3389/fpls.2026.1786995 (PMC13002568; doi:10.3389/fpls.2026.1786995)

# Variations in composition of fungal functional groups inferred by FUNGuild

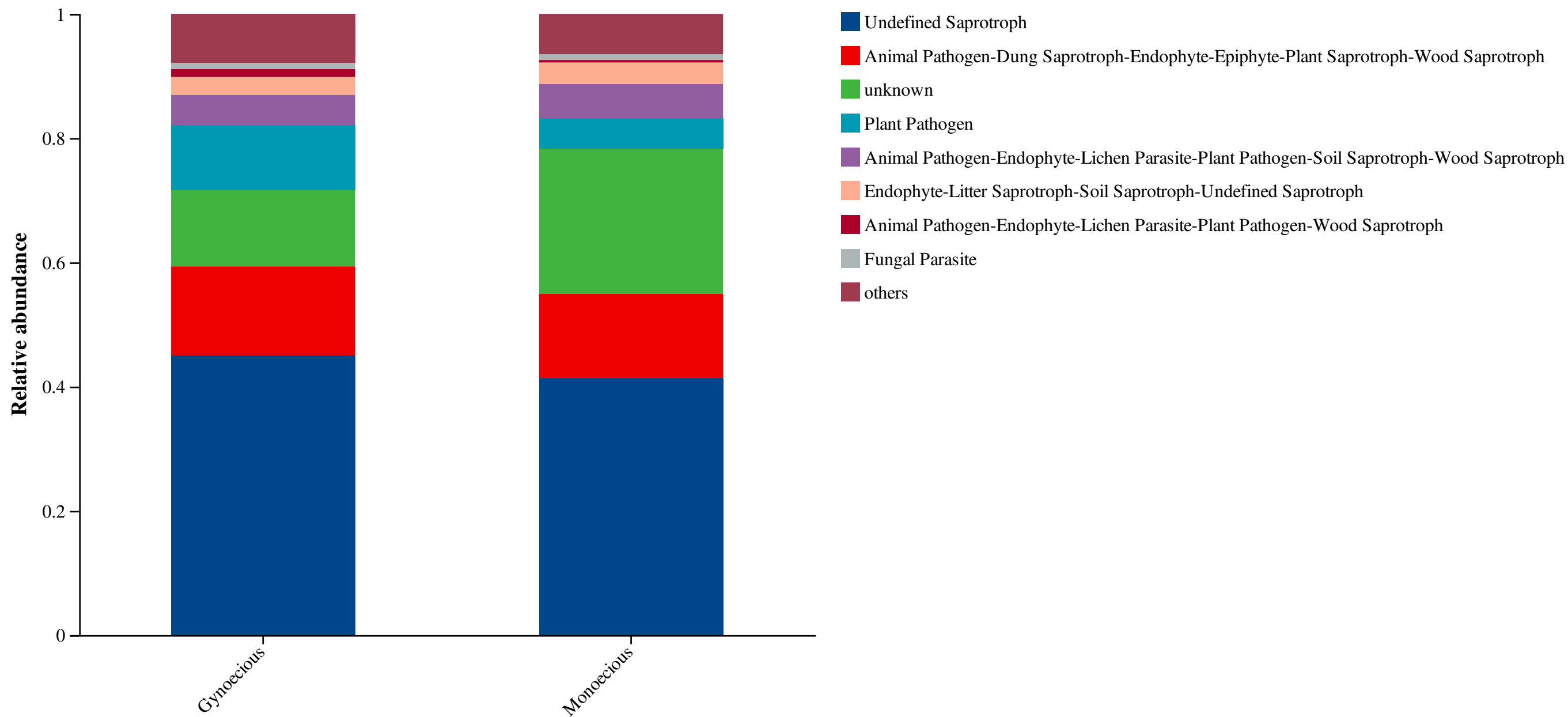

Supplement: Supplementary file 1 [file DataSheet1.zip › FUNGuild/FUNGuild_count_bar.pdf]
